# Optimal Timing of Treatment Initiation in Non-Metastatic Castration-Resistant Prostate Cancer Based on PSA Level and Doubling Time for Prognostic Benefit

**DOI:** 10.3390/cancers17223641

**Published:** 2025-11-13

**Authors:** Takuto Ogasawara, Kohei Hashimoto, Tetsuya Shindo, Ko Kobayashi, Toshiaki Tanaka, Fumimasa Fukuta, Genki Kobayashi, Ryuichi Kato, Shintaro Miyamoto, Yasuharu Kunishima, Naoya Masumori

**Affiliations:** 1 Department of Urology, Sapporo Medical University School of Medicine, Sapporo 060-8543, Japan; t.ogasawara@sapmed.ac.jp (T.O.);; 2 Department of Urology, Steel Memorial Muroran Hospital, Muroran 050-0076, Japan; 3 Department of Urology, Muroran City General Hospital, Muroran 051-0012, Japan; 4 Department of Urology, Japanese Red Cross Asahikawa Hospital, Asahikawa 070-8530, Japan; 5 Department of Urology, Sunagawa City Medical Center, Sunagawa 073-0196, Japan

**Keywords:** prostate cancer, non-metastatic prostate cancer, androgen receptor pathway inhibitors, prostate-specific antigen doubling time

## Abstract

**Background:** To clarify the timing of treatment initiation for non-metastatic castration-resistant prostate cancer (nmCRPC), we investigated the impact of baseline prostate-specific antigen (PSA) at treatment initiation on outcomes, the stability of PSADT estimation at low PSA levels, and the prognostic significance of PSADT. **Methods:** We retrospectively analyzed 129 consecutive nmCRPC patients between 2000 and 2023. All patients were divided by PSADT ≤ 10 months (*n* = 109) or >10 months (*n* = 20). PSA progression-free survival (PSA-PFS) and metastasis-free survival (MFS) were assessed by the Kaplan–Meier method, with predictive factors analyzed using Cox proportional hazards modeling. PSA-PFS was further compared across baseline PSA subgroups (<3, 3–5, 5–10, >10 ng/mL) in the PSADT ≤ 10 months cohort. **Results:** Patients with PSADT ≤ 10 months had worse MFS than patients with PSADT > 10 months (4-year: 71.9% vs. 100%; *p* = 0.021). In the PSADT ≤ 10 months group, novel androgen receptor pathway inhibitor (ARPI) treatment significantly improved PSA-PFS compared to those who did not (median: 44.0 vs. 16.6 months; *p* < 0.001). In multivariate analysis, prior definitive local therapy (Hazard Ratio [HR] 0.409, *p* < 0.001), ARPIs as first-line treatment (HR 0.421, *p* < 0.001) and lower baseline PSA at treatment initiation (HR 0.961, *p* = 0.004) were significantly predictive factors for PSA-PFS. PSADT estimation remained accurate when calculated from PSA nadir values ≥0.5 ng/mL. **Conclusions:** In patients with nmCRPC with PSADT ≤ 10 months, early initiation of ARPIs at lower PSA levels was associated with improved PSA-PFS. PSADT stabilized at PSA levels of >0.5 ng/mL. These findings support earlier ARPI initiation to optimize outcomes in high-risk nmCRPC.

## Simple Summary

In non-metastatic castration-resistant prostate cancer, the optimal timing of treatment initiation is unclear, particularly the reliability of prostate-specific antigen doubling time (PSADT) at low prostate-specific antigen (PSA) levels and the effect of baseline PSA at treatment start on outcomes. We aimed to evaluate whether earlier use of novel androgen receptor pathway inhibitors (ARPIs) is beneficial by using PSA at initiation and PSADT. We found that patients with PSADT ≤ 10 months developed metastasis more often, and starting treatment at lower PSA was linked to longer prostate-specific antigen progression-free survival. Estimation of PSADT was stable when calculated at PSA ≥ 0.5 ng/mL. These findings support earlier ARPI initiation to optimize outcomes in high-risk nmCRPC.

## 1. Introduction

The incidence of prostate cancer is increasing, and in 2019, it was the most commonly diagnosed cancer among men in Japan. Radical treatments, such as surgery and radiation therapy, are widely performed for clinically localized prostate cancer [1]. However, some patients who receive definitive local therapy eventually develop biochemical recurrence, characterized by a rising PSA level [2,3]. These patients are typically treated with androgen deprivation therapy (ADT) [4,5,6,7]. In certain cases, however, PSA levels continue to rise despite maintaining castrate levels of testosterone, indicating progression to castration-resistant prostate cancer (CRPC) [8].

Patients who exhibit no evidence of metastases based on conventional imaging and elevated prostate-specific antigen (PSA) values despite castrate levels of serum testosterone (≤50 ng/dL) after definitive treatment or systemic therapy are considered to have non-metastatic castration-resistant prostate cancer (nmCRPC) [9,10]. It has been shown that patients with nmCRPC and a short PSA doubling time (PSADT) have an increased risk of developing bone metastases and cancer mortality [10,11]. In the SPARTAN, PROSPER, and ARAMIS trials, patients with nmCRPC and PSADT ≤ 10 months treated with novel androgen receptor pathway inhibitors (ARPIs) exhibited improved metastasis-free survival (MFS) and overall survival (OS) compared with androgen deprivation therapy (ADT) [12,13,14,15,16,17]. In accordance with the results of the Advanced Prostate Cancer Consensus Conference 2022, most urologists currently recommend the use of ARPIs for nmCRPC with a PSADT value ≤ 10 months in Japan [18]. However, it is not clear when ARPI treatment should be initiated in these high-risk nmCRPC with PSADT ≤ 10 months. The clinical impact of initiating treatment at lower PSA levels—though still within the eligibility range of clinical trials—remains insufficiently explored in high-risk nmCRPC patients.

To clarify the timing of treatment initiation for nmCRPC, we assessed the correlation between PSA levels and treatment initiation, as well as PSADT and prognosis. In addition, we assessed a treatment initiation time for nmCRPC with PSADT > 10 months.

## 2. Methods

We retrospectively reviewed 129 patients consecutively admitted at our institution and associated institutions and diagnosed with nmCRPC between 2000 and 2023. All procedures involving patient information in this study were performed in accordance with the relevant guidelines and regulations. Due to the retrospective nature of the study, the Sapporo Medical University Institutional Review Board waived the need to obtain informed consent (Approval No. 302-66). All diagnoses of prostate cancer were confirmed by histopathologic examination. CRPC was defined by disease progression despite castrate levels of serum testosterone (<50 ng/dL). All patients were diagnosed with nmCRPC, “M0” in what is known as the TNM classification, by employing computed tomography (CT) and a bone scan using 99mTc-methylene-diphosphonate (bone scintigraphy) to identify soft tissue and bone metastases, respectively. Prostate-specific membrane antigen positron emission tomography (PSMA PET) was not available during the study period, and no patients underwent PET imaging. For patients with nmCRPC, the initiation of ARPIs or antiandrogen (bicalutamide or flutamide) treatment in combination with ADT was determined by the physician. In this study, ‘ARPI’ refers exclusively to second-generation androgen receptor pathway inhibitors (abiraterone, apalutamide, enzalutamide, darolutamide). First-generation antiandrogens such as bicalutamide and flutamide were analyzed separately, given the broader inhibitory profile and higher binding affinity of second-generation ARPIs.

PSADT was defined as PSA from the time of nadir after ADT to the diagnosis of nmCRPC using a PSA of at least three points to calculate PSADT, and PSADT was measured (http://www.ne.jp/asahi/akira/imakura/C_Doubling_Time.htm (accessed on 10 October 2024)). Based on the three pivotal studies defining high-risk nmCRPC as PSADT ≤ 10 months, all patients were divided into two groups according to PSADT: PSADT ≤ 10 months and PSADT > 10 months [12,13,14,15]. To clarify the stability of PSADT, we evaluated the accuracy of PSADT calculated from nadir PSA value within the defined PSA range for PSADT ≤ 10 months at the time of nmCRPC diagnosis.

The primary outcome was PSA progression-free survival (PSA-PFS). Metastasis-free survival (MFS), cause specific survival (CSS), and overall survival (OS) were also assessed as secondary outcomes. We applied multivariable Cox proportional hazards regression to identify independent predictors of PSA-PFS. PSA progression followed the Prostate Cancer Working Group 3 definition [19]. PSA-PFS was defined as the time from the initiation of treatment to the first documented PSA progression. Patients who died without PSA progression were censored at the time of death. PSA-PFS, MFS, and OS were evaluated using the Kaplan–Meier method, and statistical significance was assessed with the log-rank test used to evaluate differences between groups. To evaluate the relationship between PSA-PFS and baseline PSA levels at treatment initiation in patients categorized as the PSADT ≤ 10 months group, we sub-divided them according to baseline PSA levels (<3, 3–5, 5–10, >10, ng/mL) and calculated the 2-year PSA-PFS for each treatment. In all analysis, the *p*-value of <0.05 was defined as statistically significant. In the statistical analysis, we performed JMP Pro 17 (SAS Institute; Cary, NC, USA) and GraphPad Prism (version 10.6.1, GraphPad Software, Boston, MA, USA).

## 3. Results

### 3.1. Patient Characteristics

At the time of the initial diagnosis of prostate cancer, the cohort had a median age of 71 years and a median PSA level of 22.0 ng/mL, and distant metastasis was identified in 13 patients (10%) (Table 1). The PSADT values of 109 patients (84%) were ≤10 months and were >10 months for 20 patients (16%). At nmCRPC diagnosis, the median PSA and PSADT were 3.0 ng/mL and 4.8 months, respectively. In the PSADT ≤ 10-month subgroup, the median PSA and PSADT at nmCRPC diagnosis were 2.4 ng/mL and 3.0 months, whereas in the PSADT >10-month subgroup, they were 4.8 ng/mL and 19.0 months (*p* = 0.310 for PSA and *p* < 0.001 for PSADT). In the PSADT ≤ 10 months group, 71 patients (64%) received ARPIs, whereas 5 patients (25%) received ARPIs in the PSADT > 10 months group (*p* = 0.003). Patients in both groups received ARPI without discontinuation.

### 3.2. The Significance of PSADT in nmCRPC for Prognosis

**Figure 1:**
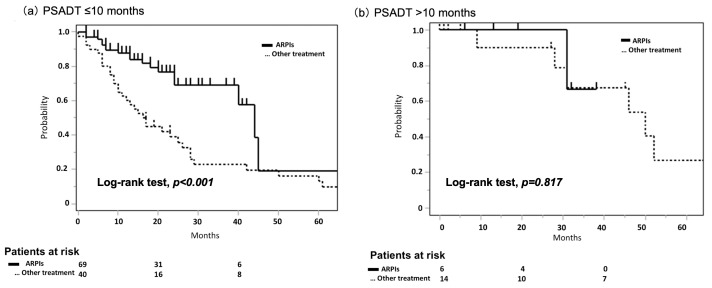
PSA progression-free survival by initial treatment (ARPIs vs. other treatments). (**a**) Patients with PSADT ≤ 10 months, (**b**) patients with PSADT > 10 months. ARPIs: androgen receptor pathway inhibitors; PSADT: PSA doubling time.

During a median follow-up time of 88 months, PSA failure, metastasis development, and all-cause mortality were observed in 62 (48.0%), 21 (16.2%), and 22 patients (17.1%), respectively. There were significant differences in the MFS between the PSADT ≤ 10 months and the PSADT > 10 months groups (4-year: 71.9% vs. 100.0%; *p* = 0.021), but not in PSA-PFS, CSS, and OS values for these groups (4-year: 28.4% vs. 54.3%; *p* = 0.110, 83.5% vs. 100.0%; *p* = 0.189, and 82.8% vs. 100.0%; *p* = 0.149, respectively). In the PSADT ≤ 10 months group, PSA-PFS was significantly prolonged in patients who received ARPIs as a first-line treatment compared with those who did not (median: 44.0 months vs. 16.6 months; *p* < 0.001) (Figure 1a). On the other hand, in the PSADT > 10 months group, the use of ARPIs as a first-line treatment did not affect PFS-PFS (median: 50.0 months vs. not reached; *p* = 0.817), (Figure 1b). In the PSADT > 10 months, nine patients had PSA progression without metastasis, of whom four (44.4%) patients were followed by ARPI.

### 3.3. Stability of PSADT

We investigated the accuracy of PSADT values calculated from PSA nadir levels up to 3.0 ng/mL for the PSADT ≤ 10 months group at the time of nmCRPC diagnosis. The sensitivity and specificity of PSADT values calculated from PSA nadir levels up to 1.0 ng/mL, 2.0 ng/mL, or 3.0 ng/mL were high (93.5% and 83.3%, 97.6% and 81.8%, and 98.7% and 90.9%, respectively) (Supplementary Table S1).

### 3.4. PSA-PFS According to Baseline PSA Level at Treatment Initiation in Patients with PSADT ≤ 10 Months

**Figure 2:**
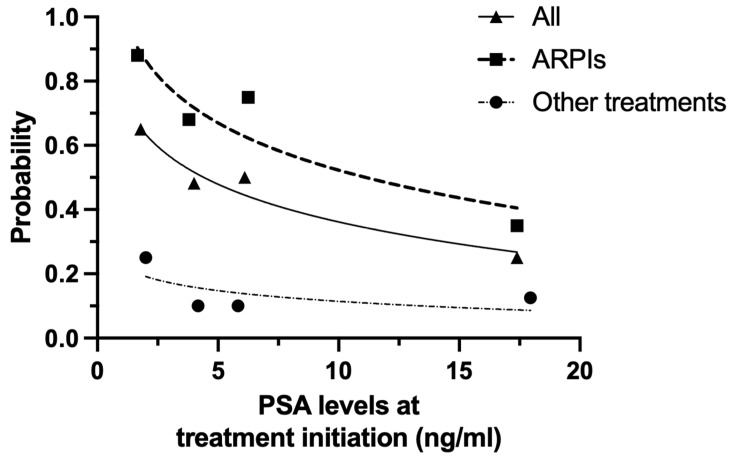
Association between PSA levels at treatment initiation and after two years of PSA progression-free survival by initial treatment (ARPIs vs. other treatments). ARPIs: androgen receptor pathway inhibitors; PSA: prostate-specific antigen.

In the PSADT ≤ 10 months group, we evaluated the association between the timing of treatment initiation and prognosis of nmCRPC. In the multivariate analysis, prior definitive local therapy (Hazard Ratio [HR] 0.409, 95% Confidence Interval [CI] 0.223–0.748, *p* < 0.001), ARPIs as the first-line treatment (HR 0.421, 95% CI 0.230–0.766, *p* < 0.001) and lower PSA at treatment initiation (HR 0.961, 95% CI 0.944–0.983, *p* = 0.004) were significantly predictive factors for PSA-PFS (Table 2). In patients with PSADT ≤ 10 months, the two-year PSA-PFS values arranged according to baseline PSA levels at treatment initiation are shown in Figure 2. The two-year PSA-PFS worsened with higher baseline PSA values at the start of treatment, regardless of whether the treatment was ARPIs or other therapy. In patients who received ARPIs, PSA-PFS in those having treatment initiation at PSA levels of ≤3 ng/mL was significantly better than in those with PSA levels of >3 ng/mL at the start of therapy (median: 44 months vs. 40 months; *p* = 0.022) (Supplementary Figure S1).

## 4. Discussion

Herein, we demonstrated that patients with nmCRPC exhibiting PSADT ≤ 10 months had worse MFS than patients with PSADT > 10 months. In the patients with PSADT ≤ 10 months, ARPI treatment improved prognosis compared to other treatments, and these data suggested that PSA levels at treatment initiation influence their prognosis. Given the stability of PSADT at PSA levels of ≥0.5 ng/mL, it is important to initiate earlier treatment with ARPIs in nmCRPC patients with PSADT ≤ 10 months.

For PSADT > 10 months, guidelines favor observation or other hormonal therapy, given the better prognosis [10,20,21,22]. In 441 nmCRPC patients, Howard et al. linked PSADT to metastasis risk [11]; median time to metastasis was 9 months for PSADT < 3, 19 for 3–8.9, 40 for 9–14.9, and 50 for ≥15 months. The median in those with PSADT ≥9 months approximated that of ARPI arms in phase III trials for cases with PSADT ≤ 10 months [12,13,14]. High PSADT reflects slow, but not halted, progression; systemic therapy effects remain unclear. In STRIVE (phase II), bicalutamide vs. enzalutamide in nmCRPC (PSADT not reported) showed PSA-PFS 11.1 months with bicalutamide and not reached with enzalutamide (*p* < 0.001) [23]. Initiating ARPIs contributed limited benefit when PSADT > 10 months, and PSA-PFS did not differ significantly. Of the patients with PSA failure treated with antiandrogen as an initial treatment, almost half were subsequently treated with ARPIs because they had PSADT ≤ 10 months at the time of PSA failure. Forty-five percent with PSADT > 10 months developed PSA failure without metastasis, suggesting vintage drugs may be less critical in this subgroup.

For nmCRPC patients with PSADT ≤ 10 months, PSMA-PET has demonstrated a high frequency of undetected metastatic lesions, [24] contributing to poor prognosis. Wang et al. reported that PSMA-PET/CT ([68Ga]-PSMA-11) results were positive for metastasis in 21 (57%) of 37 patients with nmCRPC exhibiting PSADT ≤ 10 months and a median PSA value of 0.57 ng/mL [25]. Those with shorter PSADT values were more likely to have metastasis based on the PSMA-PET results. We showed that patients with nmCRPC displaying PSADT ≤ 10 months had a higher risk of metastasis, consistent with prior studies [22,26,27]. In patients with nmCRPC exhibiting PSADT ≤ 10 months who were at high risk of metastasis, we showed that patients who started treatment with ARPIs had a significantly longer PSA-PFS than those who did not. This reinforces the role of ARPIs in delaying disease progression in high-risk nmCRPC patients.

To define optimal nmCRPC treatment timing, we analyzed PSADT stability and PSA at initiation. Phase 3 trials found ARPIs beneficial in patients with baseline PSA levels ranging from 7.78 to 11.1 ng/mL [12,13,14]; yet, the optimal start remains uncertain. PSMA-PET detects metastases in 59% at PSA 0.5–<1.0 and 75% at 1.0–<2.0 ng/mL [28]; lower PSA associates with oligometastases [29]. In the present study, PSADT estimated from PSA nadir to 0.5 ng/mL or above showed high sensitivity and specificity with respect to the reference classification of PSADT ≤ 10 months and PSA ≥ 2 ng/mL at nmCRPC diagnosis. By contrast, consistent with prior reports [30] of instability at very low PSA, PSADT estimated using only values below 0.5 ng/mL showed poor concordance with the diagnostic category. We have shown that treatment intervention at low PSA levels may contribute to improved prognosis in nmCRPC. Considering treatment intervention with ARPIs in patients with nmCRPC, it may be necessary to measure and establish a PSADT value ≤ 10 months and start treatment at PSA levels of 0.5–3 ng/mL.

There are several limitations to this study, including its retrospective design, potential biases inherent in non-randomized data, and a relatively small cohort size, which may affect the generalizability of our present findings. In particular, the large difference in the number of patients between the two PSADT groups may have influenced the results, as well as the marked difference in the number of patients who received ARPIs in each group. Additionally, in this cohort, all cases were diagnosed as conventional acinar adenocarcinoma, and no tumors showed aggressive histologic features such as small cell carcinoma or neuroendocrine carcinoma components. We also did not assess genomic alterations (e.g., *TP53*, *PTEN*, and *RB1*), so molecular aggressiveness could not be evaluated. And local progression was not systematically assessed. Prospective studies are needed to validate these findings and to establish more refined guidelines for PSA level-based treatment initiation thresholds in nmCRPC. Some patients in this study initiated ARPIs before reaching the strict PCWG3 definition of nmCRPC (i.e., PSA < 2.0 ng/mL), based on clinical judgment. While this reflects real-world practice, it may limit direct comparability with clinical trials that applied more rigid inclusion criteria. However, this study contributes to optimizing the management of nmCRPC by highlighting the prognostic relevance of PSADT and the significance of baseline PSA levels at treatment initiation with ARPIs.

## 5. Conclusions

We proposed the optimal timing of treatment in patients with nmCRPC. In patients with nmCRPC and PSADT values ≤ 10 months, treatment with ARPIs resulted in a better prognosis compared with antiandrogens. The PSADT stabilized if they displayed PSA levels of ≥0.5 ng/mL at initiation of treatment, and those with lower PSA level at treatment initiation of ARPIs exhibited a better prognosis. This suggests the need for earlier initiation of treatment with ARPIs to optimize outcomes in patients with nmCRPC exhibiting a PSADT value ≤ 10 months.

## Figures and Tables

**Table 1:** Patient characteristics.

	Overall	PSADT ≤ 10 Months	PSADT > 10 Months	*p* Value
At diagnosis				
Age, median (IQR), years	71 (66–76)	71 (66–76)	72 (66–76)	0.919
PSA, median (IQR), ng/mL	22.0 (10.6–62.1)	21.0 (9.4–60.0)	30.0 (12.1–57.8)	0.336
T stage, n (%)				0.834
≤T2	83 (64)	69 (63)	14(77)	
T3	29 (23)	25 (23)	4 (15)	
T4	17 (13)	15 (14)	2 (8)	
N stage, n (%)				0.525
N0	106 (82)	88 (81)	18 (90)	
N1	23 (18)	21 (19)	2 (10)	
M stage, n (%)				0.690
M0	116 (89)	97 (8 9)	19 (95)	
M1	13 (11)	12 (1 1 )	1 (5)	
ISUP grade, n (%)				0.626
≤3	43 (33)	35 (32)	8 (40)	
4	34 (26)	29 (27)	5 (25)	
5	41 (32)	35 (32)	6 (30)	
NA	11 (9)	10 (9)	1 (5)	
Local therapy, n (%)				0.374
prostatectomy	31 (24)	28 (26)	3 (15)	
radiation therapy ± ADT	26 (20)	23 (21)	3 (15)	
At nmCRPC diagnosis				
Time from initial ADT to nmCRPC, median (IQR), months	39 (22–83)	37 (22–74)	54 (36–92)	0.142
Age, median (IQR), years	78 (72–82)	77 (75–82)	78 (72–82)	0.181
PSA, median (IQR), ng/mL	2.8 (1.8–5.1)	3.2 (1.8–5.8)	2.4 (1.5–2.8)	0.310
PSADT, median (IQR), months	5.8 (3.1–9.1)	4.8 (3.0–7.8)	19.2 (13.0–24.0)	<0.001
First-line treatment, n (%)				
ARPIs	76 (64)	71 (64)	5 (25)	0.003
Apalutamide	12 (9)	11 (10)	1 (5)	
Abiraterone	12 (9)	11 (10)	1 (5)	
Darolutamide	41 (37)	38 (34)	3 (15)	
Enzalutamide	11 (9)	11 (10)	0	
Other treatments	53 (36)	38 (36)	15 (75)	0.003
Bicalutamide	31 (24)	19 (18)	12 (50)	
Flutamide	22 (12)	19 (18)	3 (25)	
Duration of first-line ARPIs, median (IQR), months	18 (11–25.5)	16 (11–24)	31 (16–35)	0.125

IQR: interquartile range, NA, not available, nmCRPC, non-metastatic castration-resistant prostate cancer, PSA: prostate-specific antigen, PSADT: PSA doubling time, ISUP: the International Society of Urological Pathology, ADT: androgen deprivation therapy, ARPIs: androgen receptor pathway inhibitors.

**Table 2:** Univariate and multivariate Cox proportional hazards model for PSA progression-free survival in patients with PSADT of ≤10 months.

Variables	Univariate Analysis		Multivariate Analysis	
	Hazard Ratio (95%CI)	*p* Value	Hazard Ratio (95%CI)	*p* Value
Non-metastatic disease at initial diagnosis	0.923 (0.457–2.562)	0.856		
Prior definitive local therapy	0.456 (0.255–0.815)	0.008	0.409 (0.223–0.748)	<0.001
ARPIs as a first-line treatment	0.362 (0.201–0.650)	<0.001	0.421 (0.230–0.766)	<0.001
Longer PSADT at nmCRPC diagnosis	0.973 (0.874–1.079)	0.610		
Lower PSA at treatment initiation	0.961 (0.946–0.982)	<0.001	0.961 (0.944–0.983)	<0.001

PSA: prostate-specific antigen; PSADT: PSA doubling time; ARPIs: androgen receptor pathway inhibitors; nmCRPC: non-metastatic castration-resistant prostate cancer.

## Data Availability

Data are available within the article and its Supplementary Material.

## Supplementary Materials

The following supporting information can be downloaded at: https://www.mdpi.com/article/10.3390/cancers17223641/s1, Figure S1: PSA progression-free survival by PSA levels in nmCRPC patients with PSADT ≤ 10 months who received ARPIs as a first-line treatment. Table S1: Accuracy of PSADT calculated from PSA nadir values up to 3.0 ng/mL for PSADT ≤ 10 months at the time of nmCRPC diagnosis.
