# Supplementary material for: Optimal Timing of Treatment Initiation in Non-Metastatic Castration-Resistant Prostate Cancer Based on PSA Level and Doubling Time for Prognostic Benefit
Source: Cancers (Basel). 2025 Nov 13;17(22):3641. doi: 10.3390/cancers17223641 (PMC12651908; doi:10.3390/cancers17223641)
Supplement: Supplementary file 1 [file cancers-17-03641-s001.zip › Supplementary Table S1.pdf]

Supplementary Table S1. Accuracy of PSADT calculated from PSA nadir values up to 3.0 ng/mL for PSADT ≤10 months at the time of nmCRPC diagnosis

	Sensitivity	Specificity	PPV	NPV
nadir-0.5 ng/ml	0.920	0.429	0.852	0.600
nadir-1.0 ng/ml	0.935	0.833	0.935	0.833
nadir-2.0 ng/ml	0.976	0.818	0.953	0.900
nadir-3.0 ng/ml	0.987	0.909	0.975	0.952

PSA: prostate-specific antigen; PSADT: PSA doubling time: nmCRPC, non-metastatic castration-resistant prostate cancer; NPV, negative predictive value; PPV, positive predictive value.
