# Supplementary figures and images for: Optimal Timing of Treatment Initiation in Non-Metastatic Castration-Resistant Prostate Cancer Based on PSA Level and Doubling Time for Prognostic Benefit
Source: Cancers (Basel). 2025 Nov 13;17(22):3641. doi: 10.3390/cancers17223641 (PMC12651908; doi:10.3390/cancers17223641)

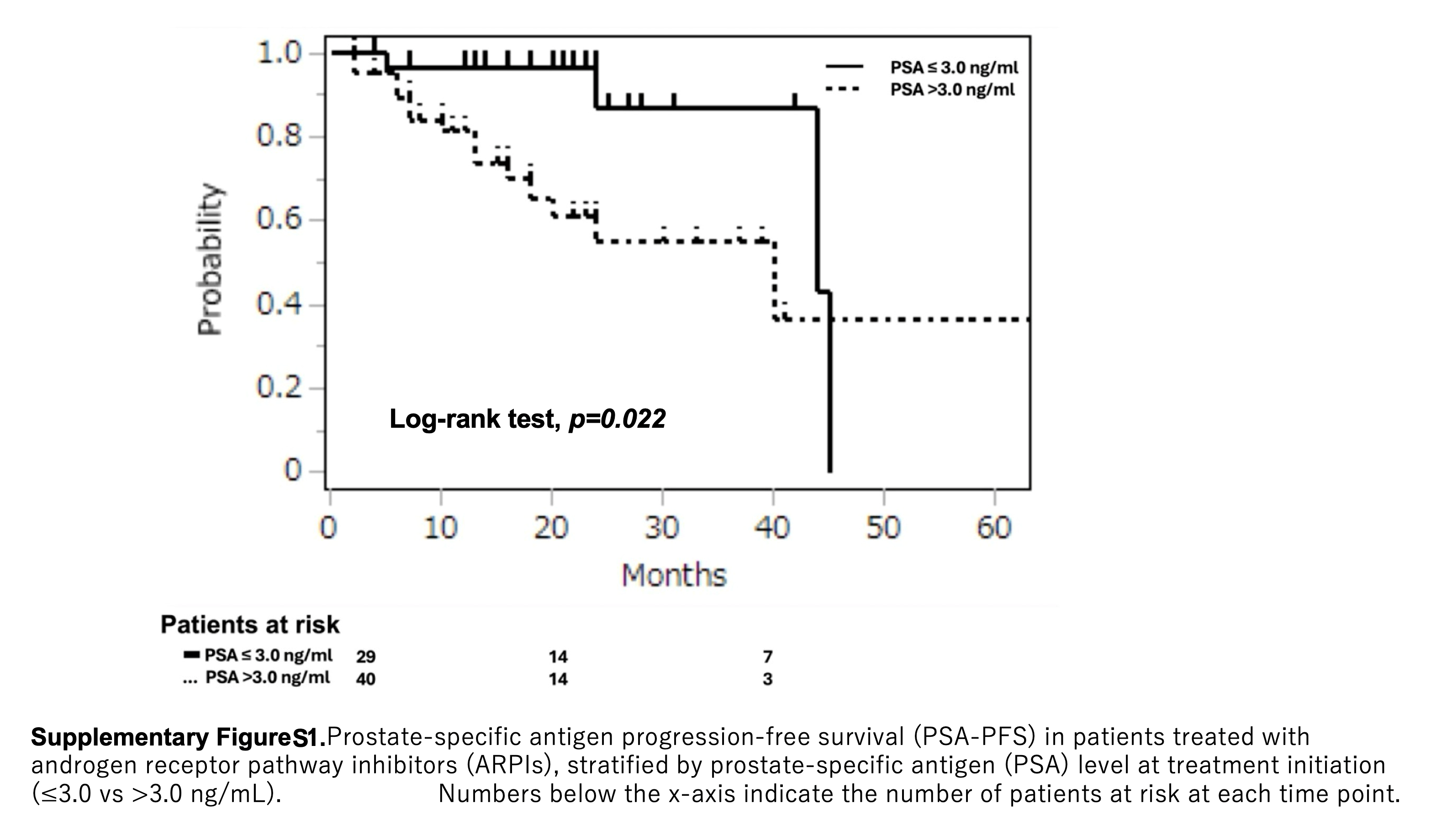

Supplement: Supplementary file 1 [file cancers-17-03641-s001.zip › Supplementary Figure S1.tiff]
